# Papillomavirus from the Bench to the Clinics

**DOI:** 10.1155/2012/437438

**Published:** 2012-03-20

**Authors:** Adhemar Longatto Filho, Luisa Lina Villa, Kari Syrjänen

**Affiliations:** ^1^Laboratory of Medical Investigation (LIM) 14, Faculty of Medicine, University of São Paulo, 01246909 São Paulo, SP, Brazil; ^2^Life and Health Sciences Research Institute, School of Health Sciences, University of Minho, 4710-057 Brago, Portugal; ^3^Institute for Cancer Research Ludwing, Hospital Alemão Oswaldo Cruz, Rua João Julião, 245–1 Andar, 01323-930 São Paulo, SP, Brazil; ^4^Department of Oncology and Radiotherapy, Turku University Hospital, Savitehtaankatu 1, 20521 Turku, Finland

Human papillomavirus (HPV) represents an exciting subject of study because it is currently established as an essential etiological factor of uterine cervical cancer and strongly implicated in the development of other genital cancers as well, in addition to benign genital warts. Additionally, substantial amount of new data have been elaborated linking HPV with head and neck cancer and, more tentatively, also with esophageal, breast, prostate, and lung cancers. Despite the existing controversies, the possible link of HPV infection with these nongenital carcinomas opens a new fascinating era of HPV research.

Concomitantly, the HPV vaccination has emerged as a new paradigm to cancer prevention programs worldwide. As much as 10% of all cancers can be related to certain HPV types, and therefore we can anticipate a substantial reduction in cancers worldwide with the implementation of HPV prophylactic vaccines. HPV vaccination provides a realistic option to reduce cervical cancer incidence and mortality in poor and developing countries, where the secondary prevention options (i.e., the screening by Papanicolaou smears and HPV testing) are not easily implemented due to lacking infrastructure, low human resources, lack of population adherence, and lack of political commitment. We can anticipate substantial reduction of HPV-related diseases.

During the past decades, HPV research has been pursued along different lines, which has resulted in an ever-increasing number of publications dissecting the multifaceted mechanisms of HPV infections and the complexity of biological cascade related to HPV-associated human carcinogenesis.

We are pleased to introduce this special issue dedicated to Papillomavirus, from the bench to the clinics, providing us an opportunity of entering this fascinating world that integrates a plethora of specialties studying the relation between this virus and human cancer.

*Adhemar Longatto Filho* *Adhemar Longatto Filho* *Luisa Lina Villa* *Luisa Lina Villa* *Kari Syrj $\ddot{\text{a}}$nen* *Kari Syrj $\ddot{\text{a}}$nen*

